# Recovery of Work-Related Stress: Complaint Reduction and Work-Resumption are Relatively Independent Processes

**DOI:** 10.1007/s10926-015-9573-6

**Published:** 2015-03-11

**Authors:** Wieke de Vente, Jan Henk Kamphuis, Roland W. B. Blonk, Paul M. G. Emmelkamp

**Affiliations:** 1 Department of Clinical Psychology, Faculty of Social and Behavioural Sciences, University of Amsterdam, Amsterdam, The Netherlands; 2 Faculty of Social and Behavioural Sciences, Research Institute of Child Development and Education, University of Amsterdam, Nieuwe Achtergracht 127, P.O. Box 15776, 1001 NG Amsterdam, The Netherlands; 3 TNO Quality of Life/Work and Employment, Leiden, The Netherlands; 4 Faculty of Social and Behavioural Sciences, Utrecht University, Utrecht, The Netherlands; 5 Netherlands Institute for Advanced Study, Wassenaar, The Netherlands; 6 The Center for Social and Humanities Research, King AbdulAziz University, Jeddah, Saudi Arabia

**Keywords:** Burnout, Fatigue, Job demands, Longitudinal study, Sick leave

## Abstract

*Purpose* The process of recovery from work-related stress, consisting of complaint reduction and work-resumption, is not yet fully understood. The aim of this study was to investigate predictors of complaint reduction and work-resumption, as well as testing complaint reduction as a mediator in the association between predictors and work-resumption. *Methods* Seventy-one patients on sickness-leave because of work-related stress complaints were followed over a period of 13 months. Predictors comprised personal (demographics, coping, cognitions), work-related (job-characteristics, social support), and illness-related (complaint duration, absence duration) variables. Dependent variables were distress complaints, burnout complaints, and work-resumption. *Results* Complaints reduced considerably over time to borderline clinical levels and work-resumption increased to 68 % at 13 months. Predictors of stronger reduction of distress complaints were male gender, less working hours, less decision authority, more co-worker support, and shorter absence duration. Predictors of stronger reduction of burnout complaints were male gender, lower age, high education, less avoidant coping, less decision authority, more job security, and more co-worker support. Predictors of work-resumption were lower age and stronger reduction of burnout complaints. No indication for a mediating role of burnout complaints between the predictor age and work-resumption was found. *Conclusions* Complaint reduction and work-resumption are relatively independent processes. Symptom reduction is influenced by individual and work-related characteristics, which holds promise for a multidisciplinary treatment approach for work-related stress.

## Introduction

Work-related stress and associated sickness absence is highly prevalent [1, 2]. Various models describe risk factors for work-related stress and its developmental mechanisms. The Job-Demand Control Support (JDCS) model of Karasek et al. [3–5], for example, states that high job-demands in combination with low job-control and/or low support elevate the risk on health problems and impaired daily functioning. Alternatively, the Transactional Model of Lazarus and Folkman [6] posits that when external demands exceed a person’s perceived ability to cope with these demands for a lasting period, health problems and impaired functioning develop. Both models state that durable exposure to high work-load can result in a state of work-related stress, which affects daily functioning and results in sickness absence. Both models are supported by substantial empirical evidence (see for example Nieuwenhuijsen et al. [7], Yu et al. [8], Lim et al. [9], and Häuser et al. [10] for reviews). Hence, substantial progress has been made in understanding the process of developing work-related stress. No less important, though far less studied, is the process of recovery from work-related stress [11, 12]. In an attempt to further enhance the insight in this recovery process, we focused on two indicators of recovery, that is, complaint reduction and work-resumption. We searched for predictors of these indicators of recovery and assessed to what extent they are related.

While complaint reduction and work-resumption are both measures of recovery, they may be affected differentially by other factors. For example, the motivation to resume work may be expected to increase with a rising risk of losing one’s job, but the risk of losing one’s job generally poses a threat, rather then promotes one’s health. As little is known about determinants of symptom reduction and work-resumption, variables regarding personal (e.g. coping), work-related (e.g. job demands), and illness-related (e.g. chronicity of the complaints) characteristics may be considered, since they have shown to be relevant in the context of work-related stress and health problems (see for example Nieuwenhuijsen et al. [7], Yu et al. [8], Lim et al. [9], and Häuser et al. [10] for reviews).

In the process of recovery from work-related stress, it may seem apparent that a reduction of complaints, or conversely, gains in health, precedes work-resumption. Accordingly, one would expect complaint reduction to predict work-resumption. Various findings suggest, however, that once absent from work, subsequent work-resumption and complaint reduction are relatively independent processes. For example, it has been shown that work-resumption frequently takes place before symptoms have reduced to normal levels [13–15], while others demonstrate that symptom reduction does not automatically result in work-resumption [16]. Also in chronic fatigue, a condition characterized by similar complaints and etiology as work-related stress, recovery and work-resumption are predicted by different variables [12]. Finally, work-resumption was successfully promoted by short cognitive behavioral interventions conducted by caregivers in the work environment (e.g., occupational physician [13, 15, 17]), while complaint reduction was not achieved by these interventions [13, 15, 17]. Thus, it remains to be tested whether complaints reduction precedes work-resumption.

In sum, this study aimed to assess the process of recovery from work-related stress by studying two aspects of recovery, that is, complaints reduction and work-resumption. This was done by identifying predictors of complaints reduction and work-resumption and testing whether complaints reduction preceded work-resumption. In order to further assess the mechanism of recovery, we assessed complaints improvement as a mediator in the association between predictors and work-resumption. Identification of predictors of recovery and/or evidence for mediation processes could provide relevant information for screening and/or treatment purposes.

For the predictors of complaints reduction and work-resumption, selection of the variables age, gender, and education was based on prediction studies targeting complaint reduction and/or work-resumption among patients absent from work because of fatigue and/or work-related stress [18–20]. Furthermore, predictors associated with the development of stress-related complaints were included. These predictors were: (a) work-characteristics as specified in the JDCS-model [4]; (b) inadequate coping, which has been associated with stress in the Transactional Model of Lazarus and Folkman [6]; and (c) dysfunctional cognitions, which are considered a risk factor for mood disorders [21]. It was assumed that more extreme values on these predictors would be associated with more severe complaints and/or less optimal conditions for recovery (e.g., low support may enhance distress). Finally, the predictors duration of complaints and duration of sickness absence were included. Duration of illness was used as an indicator of severity of complaints and/or an indirect indicator of adverse conditions for recovery (e.g., presence of an ongoing stressor such as a conflict with the employer); hence, a longer duration of either illness and/or absence duration was expected to negatively predict recovery.

Concerning the relation between complaint reduction and work-resumption, we expected at least some predictive power of complaint reduction, as a certain level of adequate daily functioning is required to be able do ones work. With respect to the mediation model, given the numerous potentially relevant predictors included, we expected to identify at least one factor that would stimulate work-resumption through complaints reduction.

This study was conducted among individuals absent from work because of work-related stress. It was part of a comprehensive project in which the effectiveness of individual and group stress-management training (SMT) was investigated. SMT did not have additional effects to care as usual on complaints or sickness absence, except for indications of superior effectiveness of individual SMT in the subgroup with lower depressive complaints [14].

## Methods

### Participants

Eighty-two patients with occupational stress were recruited through two occupational health services (*n* = 62), general practitioners (*n* = 7), and by self-referral in reaction to advertisements (*n* = 13). Eligibility was based on an intake procedure that consisted of a screening interview by telephone and a semi-structured diagnostic interview. In the screening interview, which was conducted by a clinical psychologist, presence of work-related stress complaints was examined. During the semi-structured diagnostic interview, also conducted by a clinical psychologist, the complaint history was assessed and the short version of the Composite International Diagnostic Interview (CIDI [22]) was administered. In addition, the patient filled out the Beck Depression Inventory (BDI [23]).

Inclusion criteria were: (1) fulfillment of the symptoms of neurasthenia, i.e., continuous mental and/or physical fatigue and increased fatigability, and at least two other stress complaints out of the following: dizziness, dyspepsia, muscular aches or pains, tension headaches, inability to relax, irritability, and sleep disturbance; (2) a major role of (a) work-related stressor(s) in the development of complaints, as evaluated by the referring clinician, the clinical psychologist, and the patient; and (3) presence of impaired daily functioning as indicated by (partial) sickness absence which had lasted at least 2 weeks but less than 6 months. Exclusion criteria were: (1) a primary diagnosis of major depression, social phobia, panic disorder, somatoform disorder other than undifferentiated, posttraumatic stress disorder, obsessive–compulsive disorder, hypomania, or psychotic disorders, assessed with the short version of the CIDI [22]; (2) severe depressive complaints (i.e., conservatively defined as ≥25 on the BDI [23]); (3) a traumatic event in the past 6 months; and (4) a medical condition that is commonly associated with fatigue (e.g. diabetes); (5) excessive alcohol and/or drug use; and (6) pregnancy.

### Dependent Variables

#### Distress Complaints

Fatigue was measured with the Checklist Individual Strength (CIS [24]), which consists of 20 items, divided over four subscales. Items are scored on a 7-point Likert scale ranging from 1 (false) to 7 (true). The subscale General fatigue consists of eight items. A higher score means a higher level of fatigue. Internal consistency of the subscale is generally high (e.g. [25]); Cronbach’s alpha in the current sample was also high, .91.

Depressive, anxiety, and stress-complaints were measured using the Depression, Anxiety, and Stress Scales (DASS [26]). The DASS consists of three subscales that comprise 14 items each. Severity of complaints during the past week is rated on 4-point Likert scales that range from 0 (not at all/never applicable) to 3 (very much/most of the time applicable). Higher scores represent higher levels of complaints. Psychometric properties are generally adequate to good [26, 27]. Cronbach’s alphas in the present sample were high: .87 for Anxiety, .94 for Depression, and .93 for Stress.

#### Burnout Complaints

Burnout complaints were measured with the Maslach Burnout Inventory-General Survey (MBI-GS [28]). The MBI-GS consists of 15 items regarding Emotional exhaustion (5 items), Depersonalization (4 items), and Professional competence (6 items). Items are scored on 7-point Likert scales ranging from 0 (never) to 6 (always/daily), and mean subscale scores are calculated. Higher scores indicate higher levels of work-related emotional exhaustion, depersonalization/a cynical attitude towards work, and professional competence. Reported psychometric properties are generally adequate to good [28]. Cronbach’s alphas of the subscales in the present sample were adequate to good, .85 for Emotional exhaustion, .81 for Depersonalization, and .77 for Professional competence.

#### Work-Resumption

The extent of work-resumption was assessed by self-reported hours sickness absence from work. Weekly information was obtained by using standardized diaries covering 4 weeks. Percentage sickness leave was dichotomized into ‘completely absent/partial work-resumption’ = 0, and ‘complete work resumption’ = 1.

### Predictor Variables

#### Person Related Variables

Demographic characteristics, such as age, gender, and education were assessed by questionnaire at baseline. Education level was defined as the highest completed education on a six-point scale ranging from 1 (Primary school) to 6 (University grade). Education was dichotomized in low-medium level (1–4) = 0, and high-level (5–6) = 1.

Coping was measured with the subscales Active coping (7 items) and Avoidant coping (8 items) of the Utrecht Coping Scale (UCL [29]). Items are scored on four-points scales ranging from 1 (seldom/never) to 4 (very often). Higher scores indicate a stronger tendency of using active, or confronting and avoidant, or passive coping strategies. Psychometric properties of these subscales are adequate [30]. Internal consistencies in the current sample were adequate to good: Cronbach’s alphas were .81 for Active coping, and .77 for Avoidant coping.

Dysfunctional attitudes were measured with the Dysfunctional Attitude Scale (DAS-24 [31]). The DAS-24 consists of three subscales: Achievement, Dependency, and Self-control, referring to absolutist or perfectionist beliefs about achievement, interpersonal relationships, and self-control, respectively. Items are scores on 7-points Likert scales and subscales consist of eight items each. Higher scores are indicative of stronger beliefs and more extreme scores are thought to reflect a cognitive vulnerability for psychopathology, in particular depression. Psychometric properties are adequate to good [31]. Cronbach’s alphas in the current sample were adequate to good, .89 for Achievement, .80 for Dependency, and .73 for Self-control.

#### Work-Related Predictors

The amount of official working hours at baseline was assessed by a single question about the official hours of employment.

Work-related psychosocial factors were measured by means of the Job Content Questionnaire (JCQ [32]). Items are scored on four-point scales ranging from 1 (totally disagree) to 4 (totally agree). Subscale scores of Psychological job demands, Physical exertion, Decision authority, Skill discretion, Supervisor support, and Co-worker support were calculated according to Karasek et al. [33]. Job security was based on two items (‘My job security is good’; ‘How likely is it that during the next couple of years you will lose your present job?’), the latter being scored on a three-point scale. For calculation of the Job security total score, the former item was rescaled to a three-point scale and the latter was inversely recoded. Psychological job demands and physical exertion measure different types of workload. Decision authority and skill discretion measure two aspects of job control, or decision latitude. Supervisor support and co-worker support tap two types of social support. Job security is an extra measure of a specific work-related stressor. Higher scores indicate higher levels of psychological job demands, physical exertion, decision authority, skill discretion, supervisor support, co-worker support, and job security. Psychometric properties of the subscales are generally adequate to good [33, 34]. Cronbach’s alphas in the present sample were .82 for Psychological job demands, .84 for Physical exertion, .76 for Decision authority, .78 for Skill discretion, .84 for Supervisor support, .68 for Co-worker support, and .84 for Job-security.

#### Illness-Related Predictors

Duration of absenteeism was calculated from the start of the episode of absenteeism during which the participant was included in the study. Therefore, duration of sickness absence at baseline was added to duration of sickness absence during the study. Duration of sickness leave at baseline was assessed with a single question about the duration of absenteeism. Duration of sickness leave during the study was measured using standardized diaries covering 4 weeks, in which the extent of sickness absence was reported in hours per week.

Duration of complaints at baseline was measured with a single question with the following response categories: a) <3 months, b) >3 and <6 months, c) >6 and <12 months, d) >12 months). Complaints duration was dichotomized into non-chronic duration, i.e., <6 months, and chronic duration, i.e., >6 months. This categorization is for example consistent with the criterion to discriminate adjustment disorder from undifferentiated somatoform disorder [35].

### Procedure

The ethics committee of the Department of Psychology, University of Amsterdam, approved the research protocol and all participants signed informed consent. Questionnaire data were collected five times: at baseline (T0), at the end of the treatment phase (at 4 months; T1), and at three follow-up occasions (at 7, 10, and 13 months after baseline; T2–T4). Data on sickness absence were collected using the monthly diaries during the full research period of 13 months. Extensive information about the project’s procedures, the treatment content, and the definition of care as usual is provided elsewhere [14].

### Statistical Analysis

To reduce the number of outcome measures, the seven subscale scores of distress and burnout complaints measured at baseline (Professional competence recoded inversely) were subjected to a factor analysis (oblique rotation). Examination of the Eigenvalues revealed two factors with a value above 1.00. Factor I (Eigenvalue: 3.26) consisted of Fatigue (rotated loading: .74), Anxiety, (rotated loading: .87), Depression (rotated loading: .87), and Stress (rotated loading: .87). Factor II (Eigenvalue: 1.42) comprised Emotional exhaustion (rotated loading: .64), Depersonalization (rotated loading: .88), and Professional competence (rotated loading: .73). The two factors can be interpreted as distress and burnout complaints, respectively. Composite scores for Distress and Burnout complaints were created by summing up *z*-scores of individual complaints (inversely recoded for Professional competence). In order to be able to detect change between measurements, *z*-scores were calculated per complaint for all data of all measurements at once.

Predictors that were bi-variately associated with complaints or work-resumption (*p* < .20) were entered in the regression models. Multiple longitudinal regression analyses were performed to identify baseline predictors for change of complaints (linear regression), and for change of sickness absence (logistic regression), separately. Therefore, auto-regression models were analyzed, in which each dependent variable at time T was predicted by itself at time T-1 [36]. Predictors were eliminated in a backwards procedure until the model consisted of significant predictors only (*p* < .05). Coefficients were adjusted for treatment condition. For work-resumption, first baseline predictors were included and eliminated, followed by improvement of distress and burnout complaints. Accordingly, a potential mediating role of complaint improvement could be investigated. Regression analyses were conducted with Generalized Estimating Equations (GEE; [37]) in SPSS 20.0. An exchangeable correlation matrix was used to adjust for the dependency of observations. As no collinearity diagnostics are implemented for GEE in SPSS 20.0, we inspected bi-variate correlations between predictors to detect potential collinearity.

## Results

### Descriptive Results

Data of 71 participants were available on baseline characteristics and at least two consecutive measurements on either complaints or sickness absence. Per measurement numbers of participants with valid data on any complaint and/or sickness absence were 71 at T0, between 58–70 at T1, 45–63 at T2, 45–61 at T3, and 45–60 at T4. Sample characteristics at baseline concerning predictors are presented in Table 1. In Table 2, descriptive statistics of complaints and work-resumption are listed.

**Table 1:** Descriptive information of baseline-predictors (*N* = 71)

Predictors	*n*	%	*M*	*SD*
*Person*-*related*				
Gender (0 = female, 1 = male)	41/30	58/42	–	–
Age (years)	–	–	41.61	9.48
Education (0 = low/medium, 1 = high)^a^	44/27	62/38	–	–
Active coping (7–28)	–	–	19.37	3.75
Avoidant coping (8–32)	–	–	16.11	3.98
Achievement (8–56)	–	–	28.55	10.50
Dependency (8–56)	–	–	30.99	8.85
Self-control (8–56)	–	–	33.96	7.21
*Work*-*related*	–	–		
Working hours (official hours/week)	–	–	36.14	5.19
Psychological job demands (9–36)	–	–	26.55	4.70
Physical exertion (4–16)	–	–	7.52	2.81
Skill discretion (6–24)	–	–	18.14	3.39
Decision authority (3–12)	–	–	8.57	2.11
Supervisor support (4–16)	–	–	8.89	2.61
Co-worker support (4–16)	–	–	11.31	1.97
Job-security (2–6)	–	–	4.52	1.29
*Illness*-*related*				
Absence duration (weeks)	–	–	9.07	7.76
Complaints duration (0 = non-chronic, 1 = chronic)	33/38	46/54	–	–

^a^low/medium = 1–4, and high = 5–6 on a 6-point scale ranging from 1 = Primary school–6 = University

**Table 2:** Descriptive information of dependent variables over the course of 13 months

Outcome (range)	T0		T1		T2		T3		T4	
	*M*	*SD*	*M*	*SD*	*M*	*SD*	*M*	*SD*	*M*	*SD*
Fatigue (8–56)	42.41	9.47	29.21	12.49	30.51	12.30	26.09	12.23	25.60	12.24
Anxiety (0–42)	8.36	6.80	3.78	5.65	5.68	6.19	4.83	6.26	4.92	6.69
Depression (0–42)	12.84	8.28	5.56	6.56	6.97	7.35	5.56	6.27	5.89	7.59
Stress (0–42)	18.68	8.78	8.79	7.50	11.93	7.83	10.65	8.11	10.17	8.41
Emotional exh. (0–6)	4.17	1.22	2.65	1.53	2.84	1.61	2.47	1.60	2.21	1.52
Depersonalization (0–6)	2.93	1.42	2.27	1.41	2.43	1.47	2.09	1.55	2.14	1.44
Prof. competence (0–6)	3.79	1.04	3.97	0.96	4.00	1.13	3.99	1.12	4.25	1.09

	n	%	n	%	n	%	n	%	n	%
Work-resumption (0–1)	0	0	27	39	34	54	38	61	41	68

Emotional exh. = emotional exhaustion; Prof. competence = professional competence

### Prediction of Complaint Reduction

Predictors of change of complaints are presented in Table 3. Absolute bi-variate intercorrelations between predictors were <.70 for Distress complaints, and <.60 for Burnout complaints. Hence, no indications for collinearity were found. Change of Distress complaints was predicted by personal, work-related, and illness-related variables. Reduction of Distress complaints was less among females, participants employed more hours a week, participants with more decision authority, participants with less co-worker support, and participants with longer sickness absence duration. Change of Burnout complaints was predicted by personal and work-related variables. Reduction of Burnout complaints was less among females, negatively associated with age and avoidant coping, and positively associated with education. Reduction in Burnout complaints was negatively associated with decision authority, and positively with job security and co-worker support.

**Table 3:** Regression coefficients and test results of predictors of change of complaints, adjusted for treatment condition

	B	CI B	*p*
*Distress complaints* ^a^			
Gender (0 = female, 1 = male)	−0.459	−0.823 to −0.095	.013
Working hours (official hours/week)	0.051	0.021 to 0.082	.001
Decision authority	0.061	0.004 to 0.118	.037
Co-worker support	−0.090	−0.149 to −0.030	.003
Absence duration (weeks)	0.020	0.002 to 0.038	.026
*Burnout complaints* ^b^			
Gender (0 = female, 1 = male)	−0.392	−0.717 to −0.068	.018
Age	0.017	0.005 to 0.029	.007
Education (0 = low–medium, 1 = high)^c^	−0.433	−0.769 to −0.097	.011
Avoidant coping	0.044	0.013 to 0.075	.005
Decision authority	0.130	0.061 to 0.198	<.001
Job security	−0.230	−0.366 to −0.093	.001
Co-worker support	−0.096	−0.160 to −0.033	.003

Change was analyzed by including the time-varying dependent variable at T-1 as a covariate in the model. Test results of these covariates are not reported in the table

*B* unstandardized regression coefficient (of note, the dependent variables are *z*-transformed), *CI* confidence interval

^a^Full model: gender, age, education, achievement, dependency, self-control, absence duration, complaint duration, employment (hours/week), skill discretion, decision authority, psychological job demands, physical exertion, supervisor support, co-worker support

^b^Full model: gender, age, education, active coping, avoidant coping, achievement, complaint duration, employment (hours/week), skill discretion, decision authority, physical exertion, job security, supervisory support, co-worker support

^c^Low/medium = 1–4, and high = 5–6 on a 6-point scale ranging from 1 = Primary school–6 = University

### Prediction of Work-Resumption

For work-resumption, absolute bi-variate intercorrelations between predictors were <.70. Hence, no indications for collinearity were found. Age was the only baseline-predictor that was significantly associated with work-resumption with higher age having lower odds of work-resumption. After inclusion of distress and burnout complaints, age remained a statistically significant predictor, and only improvement of burnout complaints predicted work-resumption. Less reduction of burnout complaints was associated with lower odds of work-resumption. Since the odds ratio of age changed minimally (<1 %) after addition of burnout complaints, no support for mediation of the association between age and work-resumption by improvement of burnout complaints was obtained. Outcomes of the two models are presented in Table 4.

**Table 4:** Predictors of work-resumption, adjusted for treatment condition

	OR	CI OR	*p*
*Work*-*resumption*			
Model I: baseline predictors^a^			
Age	0.944	0.902–0.989	.014
Model II: baseline predictors and complaints^b^			
Age	0.938	0.898–0.980	.004
Burnout complaints^c^	0.431	0.238–0.778	.005

*OR* odds ratio, *CI* confidence interval

^a^Full model: gender, age, education, dependency, skill discretion, physical exertion, job-security, complaint duration

^b^Full model: age, distress complaints, burnout complaints

^c^The coefficient is adjusted for burnout complaints at T-1 and thus indicates a change-score

## Discussion

This study aimed to elucidate the process of recovery of work-related stress by (a) identifying predictors of reduction of work-related stress complaints and work-resumption, and (b) exploring the association between these two aspects of recovery through a mediation model among patients with work-related stress. Distress and burnout complaints reduced considerably over the 13-months period, reaching borderline clinical levels (for a definition of clinical levels, see for example [27, 28, 38]). After 13 months, work was completely resumed by 68 % of the sample. Predictors of stronger recovery of distress complaints were being a male, working less hours per week, having less decision authority, having more co-worker support, and being absent from work for a shorter period. Predictors of recovery of burnout complaints were being a male, being higher educated, being younger, having a weaker tendency for avoidant coping, having less decision authority, having more job security, and having more co-worker support. Regarding baseline predictors, work-resumption was predicted solely by age. In addition, work-resumption was predicted by a reduction of burnout complaints in the past 3 months. No evidence for substantial mediation of the association between age and work-resumption by a reduction of burnout complaints was found. Thus, while predictors of complaints reduction and work-resumption were different, the fact that reduction of burnout complaints preceded work-resumption supports at least some relatedness between complaints reduction and work-resumption.

Our results concerning predictors of work-related complaints and work-resumption were in line with studies in related fields. For example, the variables gender, age, and co-worker support were associated with stress-related complaints in the same direction as found in the current study [11, 12, 38–41]. The finding regarding decision authority was not in concordance with the JDCS model [3–5]. These inconsistent findings may support the presumed curvilinear relationship between decision authority and health assumed by Warr [42]. Furthermore, less avoidant coping has been associated with less stress complaints [39] and recovery of depression [43]. Unexpectedly, none of the dysfunctional attitudes predicted reduced complaint reduction, though mean values of the attitudes at baseline were elevated [44, 45] and irrational cognitions have shown associations with distress complaints [46]. Inclusion of treatment condition in the models was not the reason for not findings effects; analyses without treatment condition in the model resulted in similar, non-significant coefficients (results not shown). Regarding sickness absence, higher age appears to be a consistent predictor of long-term absenteeism in patients with mental health problems, adjustment disorder, or chronic fatigue [11, 19, 47].

With respect to the mediation analysis, the association between age and work-resumption was almost entirely independent of reduction of burnout complaint. Hence, more gradual work-resumption among older participants cannot be ascribed to slower complaint reduction. An explanation for this finding may be that older patients have different attitudes towards work, which may reduce their motivation to return to work. Alternatively, employers may have different attitudes towards reintegrating older employees as compared to younger ones.

Of note, this study showed that it is relevant to distinguish between distress and burnout complaints as reductions of these complaints were predicted by different variables. In addition to the common predictors sex, decision authority, and co-worker support, reduction of distress complaints was uniquely predicted by working hours and absence duration. Unique predictors of reduction of burnout complaints were education, avoidant coping, and job-security. Moreover, only change of burnout complaints was associated with work-resumption. Post-hoc analyses (results not shown) revealed that distress complaints were also associated to work-resumption but in a different manner. Instead of change of distress complaints, it appeared that a lower level of distress complaints measured 3 months earlier predicted work-resumption. This finding suggests that a more trait-like level of *less* distress predicts more recovery.

As little is known about predictors of recovery of complaints in samples with a clinical level of work-related stress, we can only speculate about explanations for the observed associations. Females, for example, frequently have more additional obligations, such as care of the household and children (e.g., [48]), which may slow down recovery as compared to males. Individuals with less education generally tend to have more additional stressors, like for example financial problems (e.g., [49]), and tend to be less healthy (e.g., [50]), which may impair their recovery. Older individuals may recover at a slower pace because of their physical limitations. Older workers indeed need more time to recover than younger workers (e.g., [51]). Regarding working hours, findings suggest that working more hours is associated with poor health, which may impair subsequent recovery. To illustrate, working hours is associated with more psychosomatic complaints (e.g., [52]), with an unhealthier life style and with more adverse physiological changes (e.g., [53, 54]). Among individuals with more decision authority, who generally have jobs with higher responsibilities, continuous worrying on their responsibilities during their absence may hinder recovery. Individuals with less job security are likely to remain distressed while absent from work due to their uncertain future, which may prevent recovery. In support of this suggestion is that job insecurity is associated with more health complaints (e.g., [55, 56]). Reporting less co-worker support may indicate conflicts with colleagues. Conflicts with co-workers may continue to affect health during absence. It has been demonstrated that conflict with co-workers is indeed associated with more health complaints (e.g., [57]) and with delayed onset of recovery of fatigue complaints [58]. A stronger tendency of avoidant coping may prevent recovery since problems at work or during absence are less likely to be adequately solved, which may result in continuation of negative affect [59]. In addition, a stronger avoidant coping style may result in unhealthier life style behaviors [59, 60] that may in their turn delay recovery. Finally, being absent for a longer time may result in less recovery due to diminished hope on a positive outcome, reduced self-confidence, reduced positive attitudes towards work, or an increased sense of detachment to the workplace.

Considering the above proposed mechanisms, various predictor variables, though clearly in need of cross-validation, are candidates for treatment purposes. Person-related variables, e.g., coping, are already involved in cognitive-behavioral treatment (CBT). Job-related variables are less easily influenced in psychological treatments aimed at the individual such as CBT. However, regarding co-worker support, employers may encourage co-workers to support an absent patient. Employers may also enhance alternative job resources such as feedback and supervisory support to facilitate a more effective coping with job demands. For example, other researchers have observed a positive association between supervisor communication and shorter absence duration [61], supporting a more active role of the supervisor in the process of work-resumption. Furthermore, influence on job-characteristics such as working hours and decision authority may be increased by more involvement of the occupational physician in the treatment-process. Occupational physicians may add to the insight in a potential misfit between the patient and his/her work, and could stimulate the employer to make certain adjustments to the working conditions. The association between the illness-related variable absence duration and general complaint recovery may also be informative for intervention purposes. Longer absence duration may lead to aggravation of certain complaints, e.g. anxiety, or loss of day structure. Patients with longer absence duration did not have more severe complaints at baseline; associations between absence duration and complaints were low (<.20) and non-significant. This finding suggests that (partial) work-resumption may be beneficial, even though complaints may not have abated completely. However, further research is required to further investigate the association between absence duration and general complaints reduction, and investigate potential beneficial effects of earlier work-resumption.

Finally, the predictors that cannot, or with great difficulty, be changed through interventions, such as gender, age, education, and job security, can be considered as indicators of groups at risk, for whom specific interventions may be designed. Other researchers, for example, have proposed a practically applicable prediction rule based on the predictors such as age and education level that occupational physicians could use in order to identify cases at risk for unfavorable outcomes [18]. However, again, since research on predictors of recovery is scarce, replication of the results is prerequisite, before actual guidelines for identifying groups at risk can be provided.

A strength of the current study is the longitudinal design including repeated measures of both complaints and work-resumption. A main limitation of this study is that participants were predominantly employees working in small and medium size companies, and willing to participate in this intervention study, limiting generalisation to other groups of employees or the self-employed.

Future research may first of all focus on replication of the current findings. Further, future studies may aim to map the processes of recovery and work-resumption in more detail, by adopting a design in which predictors and potential mediator variables are measured repeatedly. In addition, in order to enhance the insight in work-resumption, for which we identified solely one predictor other than complaints reduction, predictors reflecting more objective psychosocial characteristics may be assessed. Support for a better prediction of sickness absence by actual job demands and control rather than perceived job demands and control has been reported [62]. Additionally, care-related indicators may be included, as other researchers reported that variables such as the number of consultations of the occupational physician or other caregivers, or communication between the supervisor and the occupational physician, were associated with work-resumption [18, 61, 63].

In conclusion, this study is an initial step in analyzing the role of individual, work-related, and illness-related variables in recovery from work-related stress. It demonstrated that different predictors exist for complaint reduction and work-resumption, suggesting that complaint reduction and work-resumption are processes driven by different forces. However, the outcome that a reduction of burnout complaints preceded work-resumption illustrates that the processes of complaint reduction and work-resumption are to some extent related. Though, in need of cross validation, our results provide initial support for promoting work-resumption through targeting burnout complaints and use of a multidisciplinary treatment approach.
